# The microbiome as a systems-level regulator of immune, metabolic, neural, and endocrine signaling in cancer

**DOI:** 10.3389/fimmu.2026.1887873

**Published:** 2026-07-27

**Authors:** Jhommara Bautista, Andrés López-Cortés

**Affiliations:** Cancer Research Group (CRG), Faculty of Medicine, Universidad de Las Américas, Quito, Ecuador

**Keywords:** cancer progression, host-microbiome interactions, human microbiome, microbiome-derived metabolites, precision oncology

## Abstract

Cancer progression and therapeutic response remain highly variable across tumor types and are not fully explained by tumor-intrinsic alterations alone. The human microbiome has emerged as a systems-level regulator of cancer biology, integrating signals across immune, metabolic, neural, and endocrine axes. Microbial dysbiosis is associated with sustained inflammatory activation, genomic instability, and epigenetic reprogramming, linking microbial composition with tumor development. Microbiome-derived metabolites, including short-chain fatty acids (SCFAs), bile acids, and tryptophan derivatives, act as intermediates connecting microbial activity with host signaling networks that regulate immune cell function, metabolic pathways, neuroimmune communication, and hormonal balance. Convergence occurs through shared intracellular pathways, including NF-κB, STAT3, and WNT/β-catenin, shaping tumor initiation, progression, and therapeutic response. Microbiome-associated profiles have been proposed as diagnostic, prognostic, and predictive biomarkers, although clinical implementation remains limited by methodological variability, cohort heterogeneity, and lack of causal validation. The review integrates current evidence within a unified systems-level framework, defines mechanistic links between microbial functional outputs and host signaling pathways, and discusses microbiome-targeted strategies with relevance for precision oncology.

## Introduction

Cancer remains a leading cause of mortality worldwide, with approximately 20 million new cases and 9.7 million deaths reported in 2022, and projections reaching 35 million cases by 2050. Although significant advances have been made in early detection, targeted therapies, and immune checkpoint blockade, variability in treatment response and survival persists across tumor types. Such heterogeneity is not fully explained by tumor genomics alone and reflects the contribution of host-associated factors, including immune status, metabolic context, and microbial composition (1, 2).

The human microbiome comprises bacteria, viruses, fungi, and archaea that colonize mucosal and epithelial surfaces and collectively encode a metabolic capacity comparable to that of the host genome. In this review, the term human microbiome is used to refer to host-associated microbial communities across anatomical sites, whereas the mechanistic discussion primarily refers to gut microbiome-derived signaling unless another body site is specified. Microbial communities regulate tumor biology through defined mechanisms. Dysbiosis promotes carcinogenesis via sustained activation of pattern recognition receptors, such as Toll-like receptors, leading to NF-κB–mediated inflammatory signaling, cytokine production, and immune dysregulation. Microbial genotoxins and metabolites further contribute to DNA damage, epigenetic modification, and oncogenic pathway activation, linking microbial composition to tumor initiation and progression (3, 4).

Microbial metabolites constitute key intermediates linking microbial activity to host signaling networks. Short-chain fatty acids (SCFAs) regulate immune cell differentiation through histone deacetylase (HDAC) inhibition and G protein–coupled receptor (GPCR) signaling, influencing CD8^+^ T-cell function and regulatory T-cell balance. Secondary bile acids interact with nuclear receptors such as FXR and membrane receptors such as TGR5, modulating metabolic pathways and immune responses within the tumor microenvironment (TME). Tryptophan-derived metabolites signal through aryl hydrocarbon receptor pathways, altering cytokine production and barrier integrity. Interactions between microbial metabolism and intracellular signaling cascades regulate tumor growth and immune surveillance (5, 6).

Microbiome–immune interactions extend beyond local signaling and shape systemic antitumor responses. Commensal bacteria influence dendritic cell activation, antigen presentation, and T cell priming, thereby determining the efficacy of immune checkpoint inhibitors (ICIs). Specific taxa have been associated with improved responses to PD-1 blockade, while dysbiosis correlates with reduced therapeutic efficacy and increased immune-related toxicity. Experimental evidence demonstrates that modulation of the gut microbiome can restore sensitivity to immunotherapy, supporting a causal role in treatment response (2, 7, 8).

Regulation mediated by microbial activity extends across metabolic, endocrine, and neuroimmune systems. Microbial enzymes such as β-glucuronidase regulate circulating estrogen levels by controlling enterohepatic recirculation, thereby influencing estrogen receptor signaling and hormone-dependent tumor development (9). Diet-induced alterations in microbial composition reshape metabolic outputs, affecting inflammation, insulin resistance, and systemic signaling pathways linked to cancer risk and progression. In addition, microbiome-derived metabolites influence neuroimmune signaling through the gut–brain axis, modulating systemic inflammation and contributing to tumor progression (10, 11).

Microbiome-associated signatures have been proposed as diagnostic, prognostic, and predictive biomarkers in oncology. Microbial DNA, metabolites, and community profiles correlate with disease risk, progression, and therapeutic response; however, clinical implementation remains limited by variability in sampling, analytical methods, and validation frameworks (12). Intratumoral microbes and their metabolites further modulate the TME by altering nutrient availability, cytokine signaling, and immune cell function, linking microbial activity to tumor behavior (13).

Despite rapid advances in microbiome research, current evidence remains largely compartmentalized, with most reviews addressing immune, metabolic, neural, or endocrine mechanisms independently. Consequently, the coordinated contribution of microbiome-derived signals across these interconnected biological systems has not been comprehensively synthesized. The present narrative review integrates current evidence on the molecular mechanisms through which microbial metabolites, structural components, and host receptor-mediated signaling pathways converge to regulate tumor initiation, progression, and therapeutic response. This systems-level perspective provides a conceptual framework for understanding microbiome–host communication in cancer while highlighting implications for biomarker discovery and microbiome-informed precision oncology.

## The human microbiome as a multisystem regulatory interface

The human microbiome consists of spatially organized microbial communities distributed across anatomically distinct niches, including the gastrointestinal tract, oral cavity, skin, respiratory tract, and urogenital tract. These microbial ecosystems differ in composition and function according to their local physiological environment. Within the gut, gradients in oxygen tension, pH, and nutrient availability generate regional heterogeneity, with microbial abundance increasing toward the colon. Colonization occurs primarily along epithelial and mucosal surfaces, where continuous exposure to dietary, environmental, and host-derived factors sustains host–microbe interactions (14–16).

Connectivity between microbial niches supports continuity across body sites. The oral cavity and intestinal tract share a subset of microbial taxa, indicating physiological translocation and persistent colonization across both environments. Microbial presence extends beyond classical mucosal sites into tumor tissues, where intracellular bacteria localize within malignant and immune cells in a tumor type-specific manner. Such distribution indicates that microbial ecosystems are embedded within both physiological and pathological compartments across the organism (17, 18).

Microbiome–host communication is coordinated through an integrated network of microbial structural components, nucleic acids, metabolites, extracellular vesicles, and microbially modified host molecules that are continuously sensed by epithelial, immune, neural, and endocrine cells. Communication begins at mucosal interfaces, where microbial-associated molecular patterns (MAMPs), including lipopolysaccharide, peptidoglycan, lipoteichoic acid, flagellin, and microbial nucleic acids, are recognized by pattern-recognition receptors such as Toll-like receptors (TLRs), NOD-like receptors (NLRs), C-type lectin receptors, and cytosolic nucleic acid sensors. Activation of these receptors initiates intracellular signaling cascades that are discussed in detail in the following section and ultimately regulate cytokine production, epithelial repair, antimicrobial peptide secretion, leukocyte recruitment, and tissue homeostasis. In parallel, microbiome-derived metabolites, including SCFAs, secondary bile acids, and tryptophan-derived metabolites, signal through G protein-coupled receptors (GPR41, GPR43, and GPR109A), nuclear receptors (FXR and PXR), TGR5, and the aryl hydrocarbon receptor (AhR), thereby coupling microbial metabolism with immune, metabolic, neural, and endocrine regulation. Beyond local sensing, microbial information is propagated through circulating metabolites, cytokines, endocrine mediators, and vagal afferent signaling, enabling intestinal microbial activity to regulate distant organs without requiring systemic bacterial dissemination (19–21).

Host–microbiome interactions follow ecological principles shaped by co-adaptation, competition, and functional redundancy. Although these physiological functions emerge from coordinated microbial communities rather than individual taxa, dominant bacterial phyla make major contributions to specific host processes. *Bacillota* and *Bacteroidota* are the principal contributors to carbohydrate fermentation and SCFA production, thereby supporting host metabolic homeostasis. *Bacillota, Bacteroidota*, and *Actinomycetota* participate extensively in immune education through regulation of epithelial integrity, antigen presentation, and immune-cell differentiation. Colonization resistance results from the coordinated activity of *Bacillota, Bacteroidota, Verrucomicrobiota*, and selected *Proteobacteria*, which compete for ecological niches and nutrients, reinforce epithelial barrier function, and restrict pathogen expansion. Microbial genomes complement host genetic capacity by encoding metabolic pathways absent in human cells, supporting a meta-organism perspective in which host and microbiome function as an integrated biological system. Bidirectional communication between microbial communities and host systems occurs through metabolites, immune mediators, and neuroactive compounds that connect intestinal, endocrine, and neural pathways (22–24).

Microbiome stability reflects preservation of functional outputs despite variation in taxonomic composition. Interindividual variability is pronounced, with each individual harboring a distinct microbial profile shaped by early-life colonization and sustained through host–environment interactions. Functional convergence across different microbial communities preserves essential metabolic activities, indicating that stability is maintained primarily at the functional rather than taxonomic level. Longitudinal studies further demonstrate that microbial composition continuously adapts to physiological states, environmental exposures, dietary changes, pharmacological interventions, and disease processes, reflecting the dynamic nature of host–microbiome interactions (25, 26).

**Figure 1:**
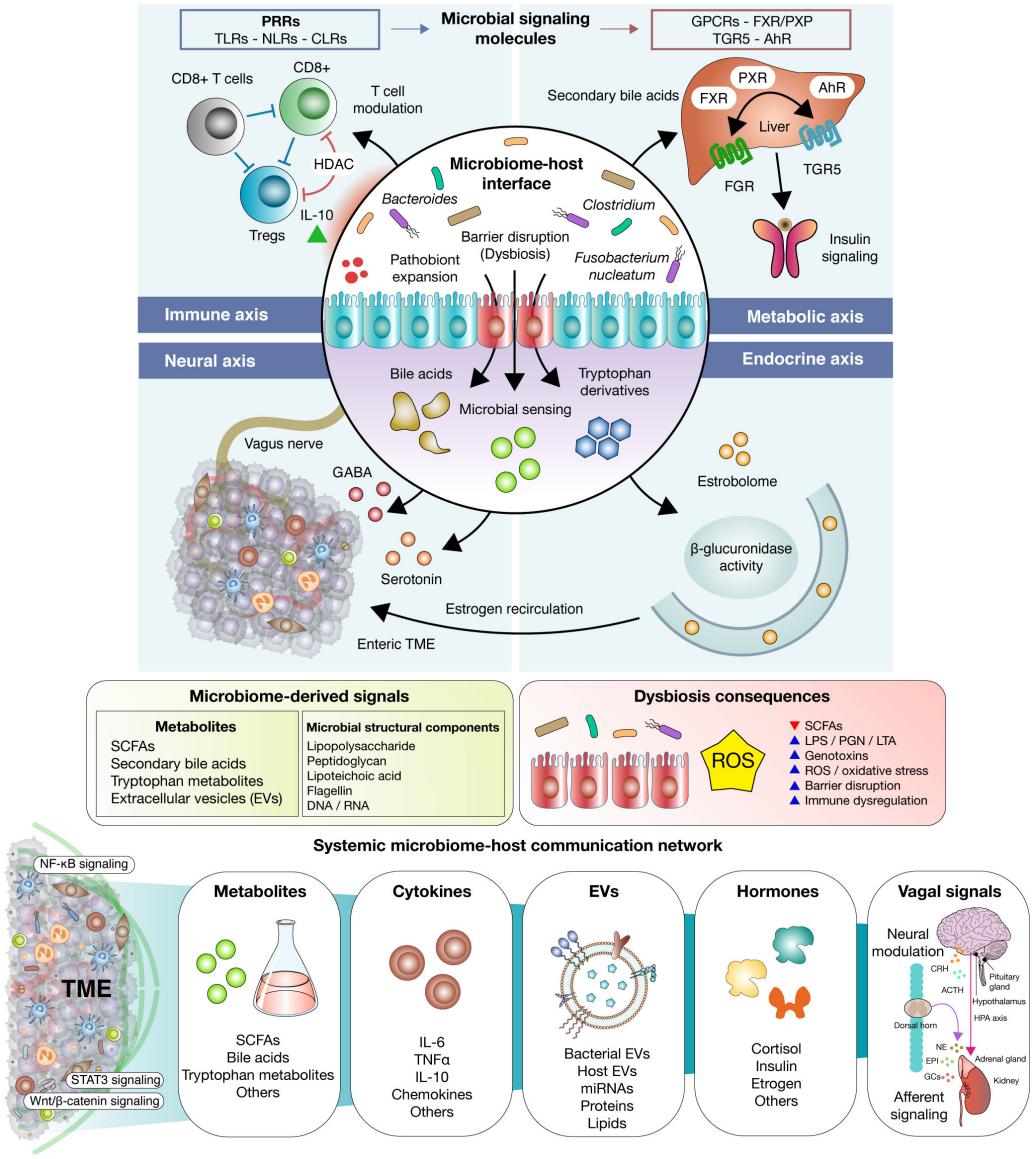
Molecular framework of microbiome–host communication in multisystem regulation. Microbiome–host communication is mediated by microbial metabolites, structural components, extracellular vesicles, and microbially modified host molecules that interact with pattern-recognition receptors, G protein-coupled receptors, and nuclear receptors. These signals coordinate immune, metabolic, neural, and endocrine axes through cytokines, circulating metabolites, hormones, extracellular vesicles, and vagal pathways, contributing to epithelial barrier integrity and physiological homeostasis. Dysbiosis disrupts this communication network by reducing beneficial microbial metabolites and increasing pro-inflammatory microbial products, genotoxins, reactive oxygen species, and barrier permeability. These alterations promote chronic inflammation, immune dysregulation, systemic dissemination of microbial signals, and tumor-supportive microenvironmental remodeling.

Dysbiosis disrupts this coordinated communication network by simultaneously altering microbial composition, metabolic output, and host receptor activation. Loss of SCFA-producing commensals reduces HDAC inhibition together with GPR41/GPR43 signaling, impairing epithelial barrier maintenance, regulatory T-cell differentiation, and anti-inflammatory programs. Concurrent expansion of pathobionts increases the release of lipopolysaccharide, peptidoglycan, genotoxins, and other pro-inflammatory molecules, resulting in sustained activation of TLR-, NLR-, and inflammasome-dependent signaling pathways. Barrier dysfunction further facilitates translocation of microbial products into the systemic circulation, amplifying cytokine production, oxidative stress, and chronic inflammatory responses. Dysbiosis also reshapes bile acid composition, perturbs microbial tryptophan metabolism, alters estrobolome activity, and modifies the production of neuroactive metabolites, thereby disrupting communication across immune, metabolic, neural, and endocrine systems. Rather than representing isolated molecular alterations, these interconnected changes progressively uncouple microbiome–host communication, compromise physiological homeostasis, and promote chronic inflammation, metabolic dysfunction, immune dysregulation, and tumor-supportive microenvironmental remodeling (**Figure 1**) (27–29).

Microbiome configuration arises from the combined influence of intrinsic and extrinsic factors. Host determinants include genetics, immune status, age, and physiological conditions, whereas external influences encompass diet, pharmacological exposure, lifestyle, and environmental context. Population-scale studies demonstrate that microbiome composition reflects the cumulative contribution of multiple variables, with no single determinant exerting dominant control over microbial community structure (30, 31).

Early-life conditions represent a critical window for microbiome establishment. Mode of delivery, antibiotic exposure, nutritional inputs, and environmental factors shape initial colonization trajectories and influence long-term microbial composition. Throughout life, continuous interactions between host physiology and environmental exposures promote adaptive restructuring of microbial communities, preserving functional resilience while allowing ecological adaptation to changing biological conditions (32, 33).

The human microbiome therefore functions as a distributed regulatory interface that integrates molecular signals across anatomical sites and physiological systems. Through coordinated ecological organization, microbial metabolism, and receptor-mediated communication, the microbiome synchronizes immune, metabolic, neural, and endocrine responses, establishing a systems-level framework that maintains physiological homeostasis and, when disrupted by dysbiosis, contributes to disease susceptibility and cancer development (34).

## Functional outputs of the microbiome in host signaling regulation

**Figure 2:**
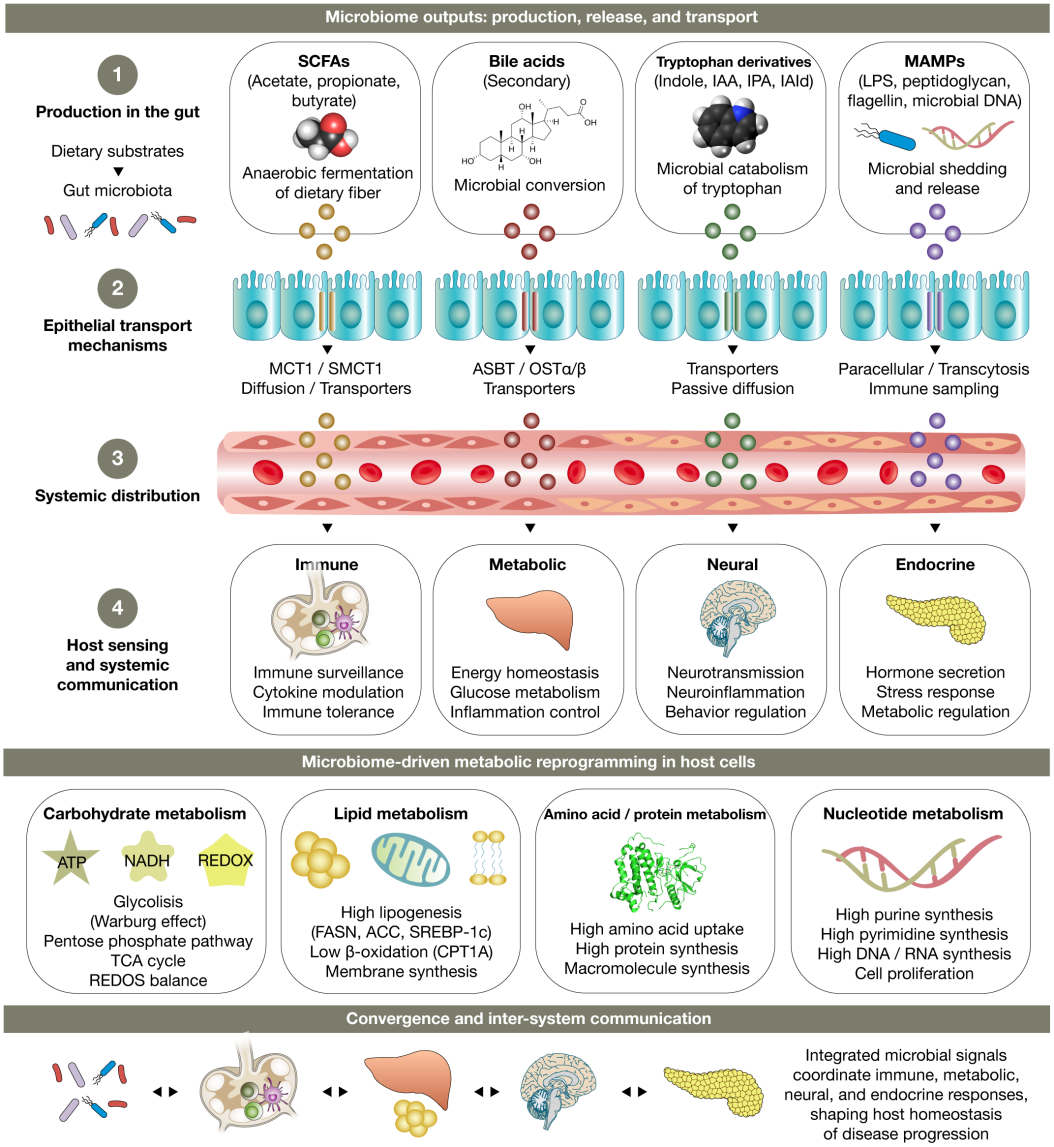
Microbiome-derived signaling molecules integrate systemic communication and host-cell metabolic reprogramming. (1) Gut microbial fermentation and co-metabolism generate short-chain fatty acids (SCFAs), secondary bile acids, tryptophan-derived metabolites, and microbe-associated molecular patterns (MAMPs). (2) Microbial products are released into the intestinal lumen and transported across the epithelium through specialized transporters, passive diffusion, enterohepatic circulation, or regulated translocation, according to their biochemical properties. (3) Following systemic distribution, microbial signals are recognized by pattern-recognition, G protein-coupled, and nuclear receptors, coordinating immune, metabolic, neural, and endocrine responses across multiple organs. (4) Microbiome-derived metabolites reprogram host-cell metabolism by modulating carbohydrate, lipid, amino acid, and nucleotide metabolic pathways, including glycolysis, the pentose phosphate pathway (PPP), and the tricarboxylic acid (TCA) cycle. Metabolic reprogramming generates ATP, NADPH, and biosynthetic intermediates required for redox homeostasis, macromolecule synthesis, cell proliferation, immune function, host homeostasis, and disease progression.

Microbiome-derived functional outputs comprise structurally diverse molecules generated through microbial fermentation, biotransformation of host-derived compounds, and endogenous bacterial metabolism. Rather than remaining confined to the intestinal lumen, many of these molecules are released, transported across the intestinal epithelium, and sensed by host cells through defined transporters and receptors, thereby enabling communication between the gut microbiota and distant organs (35, 36) (**Figure 2**).

SCFAs, principally acetate, propionate, and butyrate, are generated through anaerobic fermentation of non-digestible carbohydrates by anaerobic commensal bacteria. Following their release into the intestinal lumen, SCFAs are absorbed by colonocytes through passive diffusion in their protonated form and by the monocarboxylate transporters MCT1 (SLC16A1) and SMCT1 (SLC5A8). Most butyrate is consumed locally as the primary energy source for colonocytes, whereas acetate and propionate preferentially enter the portal circulation and subsequently reach the liver and peripheral tissues. In target cells, SCFAs activate G protein-coupled receptors GPR41 (FFAR3), GPR43 (FFAR2), and GPR109A, while butyrate also inhibits histone deacetylases. Through these complementary mechanisms, microbial carbohydrate fermentation regulates epithelial barrier integrity, immune-cell differentiation, energy metabolism, neuroimmune communication, and endocrine homeostasis (37–39).

Bile acid signaling depends on coordinated host and microbial metabolism. Primary bile acids synthesized in the liver are secreted into the intestinal lumen, where bacterial bile salt hydrolases catalyze deconjugation, followed by 7α-dehydroxylation and additional enzymatic reactions that generate secondary bile acids. Reabsorption through the enterohepatic circulation distributes primary and secondary bile acids to hepatic and extrahepatic tissues. Secondary bile acids activate nuclear receptors, including farnesoid X receptor (FXR), pregnane X receptor (PXR), and vitamin D receptor (VDR), as well as the membrane receptor TGR5, coupling microbial metabolism with regulation of lipid metabolism, glucose homeostasis, inflammatory responses, and endocrine signaling (40–42).

Microbial catabolism of dietary tryptophan generates a diverse group of indole derivatives. Intestinal bacteria convert tryptophan into indole, indole-3-acetate, indole-3-propionate, and indole-3-aldehyde, which cross the intestinal epithelium and enter the circulation. Indole-derived metabolites primarily activate the aryl hydrocarbon receptor (AhR), whereas several compounds also interact with PXR. Receptor activation regulates epithelial barrier function, mucosal immunity, cytokine production, xenobiotic metabolism, and neuroimmune signaling, providing an additional route through which intestinal microbial metabolism influences distant tissues (43, 44).

MAMPs communicate with the host through innate immune recognition rather than epithelial absorption and systemic transport. Lipopolysaccharide (LPS), peptidoglycan, lipoteichoic acid, flagellin, and microbial nucleic acids are continuously released during bacterial growth, division, and cell lysis. Recognition by epithelial and resident immune cells occurs through pattern-recognition receptors (PRRs), including Toll-like receptors (TLRs), NOD-like receptors (NLRs), C-type lectin receptors (CLRs), and cytosolic nucleic acid sensors. Under physiological conditions, an intact epithelial barrier restricts translocation, limiting signaling to local immune surveillance. Dysbiosis or barrier disruption increases passage of microbial products into the circulation, promoting inflammatory signaling in peripheral tissues and linking intestinal microbial alterations with systemic immune activation, metabolic dysfunction, neuroimmune communication, and endocrine imbalance (45–47).

Microbiome-derived metabolites and structural microbial components establish complementary routes of host communication. Diffusible metabolites support long-range signaling through epithelial transport and receptor activation, whereas MAMPs primarily trigger innate immune sensing through pattern-recognition receptors. Convergence of these routes allows intestinal microbial activity to coordinate immune, metabolic, neural, and endocrine responses across multiple organs (48).

## Microbiome-driven molecular mechanisms in cancer development

Microbiome-derived structural components, metabolites, and bacterial toxins initiate intracellular signaling through interactions with host sensing systems. LPS, bacterial adhesins, genotoxins, and microbial metabolites engage pattern-recognition receptors and intracellular sensors, linking microbial exposure to downstream signaling cascades. Activation of microbial ligands, host receptors, and intracellular pathways regulates transcriptional programs, genomic stability, and cell fate decisions associated with carcinogenesis (49, 50). All receptor-mediated signaling pathways and intracellular cascades described in this review are defined in this section to avoid redundancy across system-specific axes.

Most mechanistic evidence discussed in this section originates from colorectal cancer, where continuous exposure of the intestinal epithelium to the gut microbiota has enabled detailed characterization of host–microbe interactions. However, similar signaling pathways have also been described in other malignancies, including gastric, pancreatic, liver, breast, lung, and melanoma, although their biological relevance varies according to tissue type, microbial niche, and TME. Therefore, the pathways summarized below should be interpreted as conserved molecular mechanisms whose relative contribution is cancer type-dependent (51, 52).

Pattern recognition receptor signaling is initiated through toll-like receptors and nucleotide-binding oligomerization domain-like receptors. Lipopolysaccharide binding to TLR4 induces recruitment of adaptor proteins and activation of kinase cascades that converge on transcription factors. Experimental evidence demonstrates that *Fusobacterium nucleatum* activates a TLR4-dependent PAK1 cascade, resulting in phosphorylation and nuclear accumulation of β-catenin and induction of MYC and cyclin D1 (53, 54). In parallel, NOD-like receptor activation promotes assembly of inflammasome complexes, including NLRP3, leading to caspase-1 activation and maturation of IL-1β and IL-18. Amplification of inflammatory signaling reinforces transcriptional responses linked to tumor-promoting cellular states (55, 56).

Cytokine-mediated signaling contributes to the sustained activation of intracellular pathways after receptor engagement. Persistent microbial stimulation induces the release of IL-6 and TNF-α, promoting STAT3 and NF-κB activation. STAT3 regulates genes involved in cell-cycle progression and survival, while NF-κB coordinates inflammatory and anti-apoptotic transcriptional programs. Under chronic exposure to microbial signals, this continuous cytokine-driven activation helps stabilize oncogenic transcriptional states (57, 58). These responses are initiated by microbial sensing of structural components and metabolites. LPSs activates TLR4 signaling, whereas peptidoglycan and flagellin engage additional pattern-recognition receptors. In parallel, dysbiosis-derived secondary bile acids may further reinforce STAT3 and NF-κB activity by sustaining a pro-inflammatory TME (59).

Epigenetic regulation links microbial activity to stable transcriptional reprogramming. Microbiota-derived metabolites modify chromatin structure through histone and DNA modifications. Among SCFAs, butyrate is the principal metabolite responsible for HDAC inhibition. Following uptake by colonocytes, intracellular butyrate increases histone acetylation, modifies chromatin accessibility, and regulates transcription of genes involved in epithelial differentiation, apoptosis, and immune homeostasis. Loss of butyrate-producing bacteria during dysbiosis reduces these regulatory effects and favors a pro-tumorigenic transcriptional program (60, 61). In parallel, microbial metabolism influences the availability of methyl donors required for DNA methylation. Integrated metagenomic and epigenomic analyses have identified associations between microbial composition and altered CpG methylation patterns, including promoter hypermethylation of tumor suppressor genes and genome-wide hypomethylation associated with genomic instability. Microbial modulation of one-carbon metabolism provides a mechanistic link between microbial metabolism and long-term regulation of host gene expression (62, 63).

Oncogenic signaling pathways are activated by microbial inputs through receptor-dependent and intracellular mechanisms. Bacterial adhesins and toxins promote stabilization of β-catenin and activation of Wnt signaling independently of host mutations. Microbial stimulation of pattern recognition receptors also activates PI3K/AKT signaling through adaptor-mediated recruitment of downstream kinases, supporting cell survival, proliferation, and metabolic adaptation. Microbial genotoxins such as colibactin induce DNA double-strand breaks, contributing to mutational events that reinforce oncogenic signaling networks (58, 64, 65).

Cytosolic sensing pathways extend microbial signaling beyond membrane receptors. Microbial DNA and bacterial components entering the cytosol activate cGAS, leading to production of cyclic GMP–AMP and activation of STING. STING signaling induces interferon responses and interacts with NF-κB and MAPK pathways, integrating innate immune sensing with transcriptional regulation. Concurrent activation of ERK signaling modulates proliferation and stress-response programs, expanding intracellular responses triggered by microbial exposure (66, 67). The contribution of microbiome-associated molecular pathways varies across tumor development. In early carcinogenesis, epithelial barrier disruption, chronic inflammation, and microbial genotoxins are major factors involved in malignant transformation. As tumors progress, microbiome-derived metabolites may further support immune evasion, metabolic adaptation, angiogenesis, and therapeutic resistance by modulating NF-κB, STAT3, PI3K/AKT, and Wnt/β-catenin signaling. Therefore, the relevance of each pathway depends on tumor type, disease stage, and the biological context of the TME (67, 68).

## Immune axis microbiome-driven modulation of tumor immunity

IInnate immune modulation depends on microbiome-derived signals that regulate barrier integrity, antigen sensing, and myeloid cell activation. Disruption of epithelial tight junctions increases microbial translocation and exposure to pathogen-associated molecular patterns, sustaining inflammatory signaling and cytokine production (4). Commensal depletion and pathobiont expansion promote immunosuppressive macrophage polarization and reduce dendritic cell antigen presentation, limiting effective T cell priming. Microbiome-derived metabolites preserve epithelial integrity and regulate immune activation thresholds, maintaining controlled immune activation while preventing excessive inflammation (69). Most mechanistic evidence supporting these immune interactions has been generated in colorectal cancer, where host–microbiome interactions have been characterized most extensively. Among microbiome-derived metabolites, SCFAs, including butyrate, promote epithelial barrier integrity through GPR41, GPR43, and GPR109A signaling together with HDAC inhibition, thereby limiting excessive NF-κB activation and preserving dendritic cell function. Loss of SCFA-producing bacteria during dysbiosis weakens these immunoregulatory mechanisms, favoring chronic inflammation and immune remodeling within the TME (70, 71).

Adaptive immune regulation is shaped by microbiome-dependent control of T cell differentiation and effector function. Commensal taxa promote Th1 polarization and cytotoxic CD8^+^ T cell responses, whereas dysbiosis favors regulatory T cell expansion and suppressive cytokine profiles (72). Butyrate produced by *Roseburia intestinalis* enhances CD8^+^ T cell effector function and cytokine production, activates NF-κB signaling, and induces IFN-γ, TNF-α, and granzyme B expression, thereby increasing cytotoxic activity against tumor cells. Microbial metabolites also regulate immune-cell metabolism. In colorectal cancer, microbiota-dependent changes in amino acid availability, including microbial regulation of asparagine metabolism, impair CD8^+^ T-cell metabolic fitness, whereas butyrate supports mitochondrial metabolism and effector differentiation. This metabolic remodeling alters nutrient availability within the TME and modulates antitumor immunity. Microbial peptides can be presented via MHC class I molecules, generating cross-reactive T cell responses against tumor-associated antigens and strengthening immune recognition (73–75).

Tumor immune evasion and microenvironment remodeling involve microbiome-driven reprogramming of immune composition. Dysbiosis promotes recruitment of regulatory T cells and myeloid-derived suppressor cells, reduces cytotoxic lymphocyte infiltration, and sustains inflammatory signals that favor immune suppression. Intratumoral microorganisms interact directly with immune and tumor cells, altering cytokine gradients, metabolic conditions, and stromal interactions (76, 77). Secondary bile acids and tryptophan-derived indoles further shape local immunity. Secondary bile acids regulate macrophage polarization and dendritic-cell activation through FXR and TGR5, whereas indole metabolites activate AhR, influencing cytokine production, regulatory T-cell differentiation, and epithelial barrier function. The immunological consequences depend on metabolite availability, receptor expression, and tumor context. Systemic microbiome composition defines the threshold required for effective immune activation, linking reduced diversity with impaired immune surveillance and tumor progression (78, 79).

Microbial metabolites and community composition influence immunotherapy response by regulating dendritic-cell maturation, CD8^+^ T-cell activation, cytokine production, and immune checkpoint sensitivity. Gut microbial composition determines response to ICIs targeting PD-1, PD-L1, and CTLA-4 pathways (80). Specific taxa promote tumor-specific CD8^+^ T-cell activation and increase therapeutic efficacy. Strain-level microbial signatures are associated with treatment response and progression-free survival, supporting microbiome-based stratification. Increased microbial diversity correlates with improved immunotherapy outcomes and enhanced immune competence (81, 82).

## Metabolic axis microbiome-mediated metabolic reprogramming

Microbiome–host co-metabolism governs the biochemical transformation of dietary substrates, amino acids, and xenobiotics, generating metabolites that modulate host metabolic pathways. These products alter substrate availability and cellular energy balance, shaping systemic metabolic states. Microbial conversion of primary to secondary bile acids engages FXR and TGR5 signaling, regulating lipid metabolism, glucose homeostasis, and energy balance. In parallel, intestinal microbial enzymes transform xenobiotics and carcinogens, modifying their chemical structure, bioavailability, and systemic distribution, thereby altering exposure of distal tissues to active intermediates (9, 83).

Systemic metabolic regulation depends on microbiome-derived metabolites that circulate and influence endocrine, hepatic, and adipose pathways. SCFAs signal through GPR41 and GPR43 to regulate insulin sensitivity and energy balance, while bile acid pools modulate lipid and glucose metabolism at systemic and hepatic levels. Microbial metabolism of branched-chain amino acids and tryptophan generates intermediates that alter metabolic signaling and inflammatory tone. Circulating metabolites determine composition of tumor interstitial fluid, where nutrient availability reflects plasma metabolite levels and influences metabolic activity of tumor and immune cells (84, 85).

Beyond systemic metabolic regulation, microbiome-derived metabolites also influence central carbon metabolism in tumor and immune cells. SCFAs, bile acids, indole derivatives, and vitamin-related cofactors modify glucose uptake, glycolytic enzyme activity, and lactate production according to nutrient availability and cellular context. Altered glycolytic flux changes the supply of pyruvate and acetyl-CoA to the tricarboxylic acid (TCA) cycle, affecting mitochondrial respiration, ATP generation, and the availability of citrate, α-ketoglutarate, and succinate for biosynthetic reactions. Diversion of glucose-6-phosphate into the pentose phosphate pathway (PPP) increases NADPH production for redox homeostasis while generating ribose-5-phosphate for nucleotide synthesis. Microbiome-dependent folate, riboflavin, and one-carbon metabolism further supports purine and pyrimidine biosynthesis, DNA replication, RNA synthesis, and methylation reactions. Regulation of glycolysis, TCA cycle activity, PPP flux, and nucleic acid synthesis links microbial metabolism to anabolic capacity, redox homeostasis, and metabolic fitness within the TME (86–88).

Tumor metabolic plasticity depends on substrate availability and is directly shaped by microbiome-controlled nutrient flux. Microbial metabolism of amino acids alters concentrations of key substrates within the TME. Microbiota-encoded asparaginase reduces local asparagine levels, decreasing transport through SLC1A5 in CD8^+^ T cells, which impairs metabolic fitness, reduces stem-like properties, and limits antitumor activity. In parallel, microbiota-derived cofactors regulate host lipid metabolism; riboflavin-producing bacteria increase flavin adenine dinucleotide levels in adipose tissue, activating FADS2 and promoting polyunsaturated fatty acid synthesis, which enhances CD8^+^ T cell cytotoxic function and tumor control (9, 89).

Lipid metabolism represents a major axis of tumor metabolic reprogramming and is modulated by microbiome-driven metabolic inputs. Cancer cells increase *de novo* lipogenesis and lipid uptake to support membrane synthesis, energy storage, and resistance to therapy. Microbiome activity regulates intestinal lipid absorption, hepatic lipogenesis, and systemic lipid distribution, thereby controlling substrate availability for tumor cells. Interactions between tumor cells, stromal components, and microbiome-derived metabolites alter lipid flux within the TME, supporting proliferation, metastasis, and chemoresistance (90–92).

Metabolic disorders reinforce microbiome-driven metabolic alterations that promote tumorigenesis. Dysbiosis associated with obesity increases production of metabolites linked to inflammation, lipid accumulation, and impaired glucose regulation. Microbiome-mediated changes in energy harvest, fat storage, and insulin sensitivity elevate circulating glucose, lipids, and inflammatory mediators. Elevated nutrient availability activates signaling pathways such as PI3K–AKT and mTOR in tumor cells, increasing proliferation, survival, and metabolic demand. Obesity-associated metabolic states modify nutrient competition within the TME, altering immune cell metabolism and reducing antitumor responses (84, 93).

Microbiome-driven metabolic outputs do not operate as isolated biochemical signals but define substrate availability within the TME, constraining metabolic fluxes across tumor and immune compartments. Variation in microbial metabolite pools alters nutrient accessibility, reshaping competition between proliferating tumor cells and infiltrating immune populations. These shifts influence anabolic demand, mitochondrial function, and redox balance, ultimately determining whether metabolic conditions support tumor expansion or restrict cellular fitness under resource-limited conditions (94, 95).

## Neuro axis microbiome-mediated neural signaling

Bidirectional signaling between the gut microbiota and the central nervous system involves neural, immune, and metabolic routes. Microbial dysbiosis alters vagal afferent input, circulating metabolite pools, epithelial barrier integrity, and systemic cytokine tone, modifying neural and immune states relevant to tumor biology. In glioma, reduced SCFA-producing taxa and altered microbial composition have been associated with changes in the tumor immune microenvironment, supporting a model in which gut-derived signals influence brain tumor progression through neuroimmune pathways. Similar observations have emerged in colorectal, pancreatic, breast, and prostate cancers, where microbiota-dependent neural and inflammatory signaling has been associated with tumor-associated immune regulation, systemic stress responses, and treatment-related neurological symptoms (96, 97). Evidence for microbiome-mediated neural signaling is strongest in glioma. Studies in other tumor types remain largely preclinical and require further clinical validation (98).

Microbial metabolites constitute a major biochemical interface between intestinal microbiota and neural signaling. Variations in microbial metabolic activity modify circulating neuroactive compounds, barrier permeability, cytokine exposure, and systemic neuroimmune communication. Altered metabolite availability has been associated with changes in glial activation, neural inflammation, and tumor-associated immune states in brain malignancies. Additional microbial products, including bile acid derivatives and other neuroactive intermediates, further contribute to neural and inflammatory alterations linked to tumor progression. Neuroimmune effects associated with microbial metabolites operate through signaling pathways described in the molecular mechanisms section (99, 100).

Microbiota-dependent modulation of neurotransmitter systems connects microbial metabolism with neural circuit regulation. Gut microorganisms regulate serotonin, gamma-aminobutyric acid, dopamine-related pathways, and other neuroactive compounds that influence neuronal excitability, glial activation, and immune-cell behavior. Serotonin produced in the gut activates afferent neurons, while microbiota-driven GABA signaling alters central receptor expression through vagus-dependent mechanisms. In cancer, altered neurotransmitter signaling has been associated with modulation of tumor-relevant immune and inflammatory states. Experimental studies indicate that GABA-related signaling influences macrophage activity and CD8^+^ T-cell function, whereas serotonin-associated pathways intersect with stress adaptation, tumor progression, and treatment response across multiple tumor models. Clinical observations primarily support associations with symptom burden, neuroinflammation, and treatment-related neurological dysfunction rather than direct causal effects on tumor progression (101–103).

Neural regulation of tumor growth depends on direct innervation and receptor-mediated signaling within the TME. Tumors are infiltrated by sympathetic, parasympathetic, and sensory nerve fibers that regulate proliferation, invasion, and dissemination. Sympathetic neural activity promotes tumor initiation through β-adrenergic pathways, whereas parasympathetic cholinergic inputs have been linked to metastatic dissemination through muscarinic receptor signaling. Neural inputs also control immune-cell recruitment, macrophage polarization, and cytokine production, shaping local inflammatory conditions that influence tumor progression. Disruption of neural inputs reduces tumor development, supporting a functional role for neural circuits in oncogenesis (100, 104).

Tumor–brain communication operates through defined afferent–efferent circuits integrating peripheral tumor signals with central neural responses. Sensory neurons detect tumor-derived signals and relay information to brainstem nuclei, which increase sympathetic output to the TME. Noradrenergic signaling through β2-adrenergic receptors in immune cells promotes immunosuppressive phenotypes that limit antitumor responses. Disruption of this sensory-to-sympathetic axis restores immune activity and reduces tumor growth, establishing a mechanistic chain linking tumor sensing, central processing, and peripheral immune modulation (105, 106).

Stress-associated signaling integrates microbiome dynamics with cancer progression through neuroendocrine pathways. Activation of the hypothalamic–pituitary–adrenal axis and sympathetic nervous system increases glucocorticoids and catecholamines, altering gut permeability, microbiota composition, and immune-cell function. Glucocorticoids impair antigen presentation and promote T-cell dysfunction, while β-adrenergic signaling enhances immunosuppressive cell populations and inflammatory remodeling within tumors. In glioma models, microbiota-dependent signaling through *Bacteroides* increases systemic TGFβ, which activates microglial ERK signaling, induces *Ccl3* expression, and recruits CD8^+^ T cells that sustain tumor growth. Microbiota depletion or TGFβ neutralization disrupts this axis and suppresses tumor progression, supporting a causal role for microbiome–neural–immune interactions in oncogenesis. Although microbiome–neural–immune interactions are increasingly supported by mechanistic studies, translational evidence remains uneven across cancer types. Most causal data derive from glioma and experimental stress-associated tumor models, whereas clinical validation in broader oncologic settings remains limited and largely associative (107–109).

## Endocrine axis microbiome-mediated hormonal regulation

Microbiome-mediated endocrine regulation is most relevant in hormone-dependent malignancies. Evidence is strongest for breast and endometrial cancers, where microbial control of estrogen metabolism can modulate estrogen receptor signaling. Additional evidence has been reported in prostate cancer through microbiome-dependent regulation of androgen metabolism. Endocrine regulation by the microbiome is driven by enzymatic control of steroid hormone metabolism and reciprocal modulation between host hormones and microbial ecology. After hepatic glucuronidation and sulfation, conjugated estrogens are excreted into bile and reach the intestinal lumen, where bacterial β-glucuronidase and sulfatase enzymes catalyze deconjugation. Free estrogens released through deconjugation may undergo enterohepatic recirculation, increasing systemic estrogen availability. The estrobolome comprises microbial genes encoding these enzymatic functions, with taxa such as *Clostridium*, *Bifidobacterium*, *Roseburia*, and *Faecalibacterium* contributing to estrogen deconjugation capacity. Variation in estrobolome activity regulates the balance between fecal excretion and systemic estrogen reabsorption (110–112).

Microbial diversity and functional capacity determine endocrine output. Dysbiosis reduces β-glucuronidase activity, resulting in accumulation of conjugated estrogens in the intestinal lumen, increased excretion, and reduced circulating estradiol levels. Antibiotic-induced microbiome depletion produces similar reductions in enzymatic activity and estradiol bioavailability, establishing a direct relationship between microbial composition and hormone regulation. Higher microbial diversity is associated with increased deconjugation capacity and enhanced estrogen reabsorption. Microbial metabolism also modifies androgen precursors and circulating androgen levels through enzymatic transformations and host–microbe metabolic interactions, extending microbiome-driven regulation to multiple steroid hormone classes (113–115).

Hormonal signaling shapes microbial composition and function. Declining estrogen levels are associated with reduced microbial diversity and shifts in taxa distribution, including depletion of commensal genera and enrichment of pathobionts (116) (H2). Estrogen signaling regulates epithelial barrier integrity, immune tone, and microbial habitat conditions, which determine microbial structure. Reciprocal interactions between hormone levels and microbial metabolism connect endocrine signaling with microbial homeostasis at the systemic level (117).

Microbiome-mediated modulation of estrogen availability influences hormone-dependent cancers. Increased β-glucuronidase activity enhances estrogen deconjugation, elevates circulating estrogen levels, and increases activation of estrogen receptors in peripheral tissues, promoting proliferation in estrogen receptors–positive tumors such as breast and endometrial cancer. Reduced enzymatic activity lowers estrogen reactivation but is associated with dysbiosis, inflammation, and metabolic alterations linked to carcinogenic processes. In breast cancer, estrobolome activity regulates systemic estrogen exposure and interacts with endocrine therapy, where microbial metabolism modifies responses to aromatase inhibitors and selective estrogen receptor modulators. Longitudinal analyses show therapy-associated shifts in microbial taxa, including increases in *Blautia*, *Dialister*, and members of *Lachnospiraceae* (111, 117, 118).

Altered estrobolome activity contributes to hormone-dependent non-malignant conditions. Dysregulated β-glucuronidase activity is associated with increased local estrogen reactivation, elevated inflammatory cytokines, and sustained immune activation in endometriosis and infertility, linking microbial estrogen metabolism with chronic inflammatory and proliferative disorders (119).

Microbiome–endocrine interactions extend to immune regulation in cancer. Modulation of sex hormone levels by the microbiome may contribute to altered ICI responses through effects on CD8^+^ T cell activity and tumor immunity. External factors, including diet and long-term pharmacological treatments, modify microbial taxa associated with β-glucuronidase and sulfatase activity, altering estrogen metabolism and systemic hormonal balance. Disruption of microbiome-driven endocrine regulation affects hormone availability, receptor activation, immune signaling, and disease progression, linking microbial metabolism with endocrine and oncological outcomes (120, 121).

## Systems-level integration of microbiome-driven signaling

The relative contribution of immune, metabolic, neural, and endocrine signaling differs among tumor types. Gastrointestinal cancers provide the strongest evidence for immune and metabolic regulation, glioma provides the strongest evidence for neural regulation, and breast, endometrial, and prostate cancers are mainly shaped by endocrine mechanisms. Despite these differences, shared intracellular signaling pathways integrate microbiome-derived signals across cancer types. Microbial communities generate metabolites, structural components, and enzymatic activities that act locally within tissues or systemically after entry into circulation, influencing immune cell function and tumor cell behavior (122, 123). Microorganisms present in tumor niches and peripheral sites further contribute to signaling integration by modulating proliferation, immune activation, and tissue organization through direct molecular interactions and metabolite-mediated signaling (57, 124).

Crosstalk across systems is mediated by microbiome-derived functional outputs acting through signaling pathways defined in the molecular mechanisms section. At the systems level, integration reflects coordinated effects across immune, metabolic, neural, and endocrine axes rather than independent pathway activation. Microbiome-associated signals propagate through interconnected host networks that collectively regulate inflammatory tone, metabolic adaptation, neuroimmune communication, endocrine balance, and stromal organization within tumor ecosystems. Systemic signaling integration therefore emerges from coordinated host–microbiome interactions distributed across tissues, circulation, and tumor-associated microenvironments (2, 4, 125).

Endocrine integration contributes to system-level regulation by linking microbial metabolic activity with host hormonal signaling networks. Variability in microbiome composition influences endocrine homeostasis, metabolic balance, and inflammatory states across multiple tissues, generating interindividual differences in tumor-associated signaling environments. Dysbiosis-associated alterations in systemic communication networks further contribute to coordinated changes in immune regulation, metabolic adaptation, and tissue-specific responses relevant to tumor progression and therapeutic variability (117, 126).

Convergence arises from shared intracellular pathways previously described, integrating inflammatory, metabolic, proliferative, neural, and endocrine signals into unified tumor phenotypes. Coordinated signaling across multiple biological systems generates interconnected effects on immune organization, metabolic plasticity, stromal remodeling, angiogenesis, and therapeutic response. Rather than functioning as isolated pathways, microbiome-associated signals operate through dynamic network interactions that collectively shape tumor behavior and microenvironmental adaptation (2, 127).

System-level integration is supported by multi-omics analyses that resolve coordinated host–microbiome networks. Integrated microbiome–metabolome–immune profiling identifies associations between microbial taxa, metabolic pathways, and immune cell states, where amino acid and lipid metabolism correlate with T cell infiltration and therapeutic response. Network-based analyses reveal structured interactions linking microbial abundance to metabolite production and host signaling pathways, demonstrating that microbiome-driven effects propagate through interconnected metabolic and immune circuits rather than isolated mechanisms. Computational modeling of dysbiosis indicates that network topology and functional connectivity determine signaling propagation and system-level behavior, influencing treatment response and disease progression (58, 128, 129).

Emergent properties arise from the collective behavior of microbial communities and interaction networks with host systems. Functional outputs depend on community composition, metabolic redundancy, and ecological interactions, where reduced diversity or expansion of pathobionts alters global signaling balance and disrupts coordinated host responses across tissues. Resulting alterations contribute to persistent inflammatory states, impaired immune regulation, metabolic disequilibrium, and progressive microenvironmental instability associated with tumor progression. Context-dependent effects generated through interconnected signaling networks further support nonlinear biological responses that vary according to tissue context, ecological structure, and host physiological state (19).

Integration follows a hierarchical organizational structure in which microbiome composition determines functional outputs that subsequently influence interconnected host signaling systems. Coordinated communication across immune, metabolic, neural, and endocrine axes generates system-level responses that shape tumor phenotypes, including immune evasion, metabolic adaptation, sustained proliferation, angiogenesis, and therapeutic resistance. Functional consequences therefore emerge from network-wide interactions rather than isolated molecular events (57, 130).

## Clinical implications of microbiome-driven signaling

Intratumoral and extra-tumoral microbial communities have been associated with cancer detection through compositional and functional signatures derived from fecal, oral, and tissue samples. Metagenomic analyses identify cancer-type–specific microbial patterns that distinguish malignant from non-malignant states and capture intratumoral heterogeneity. Oral microbiome profiling coupled with machine learning generates diagnostic classifiers with high accuracy in head and neck cancer, based on species abundance and metabolic gene content (131, 132). Within tumor tissues, microbial localization correlates with activation states of signaling pathways described in the molecular mechanisms section, linking microbial detection with functional tumor-associated processes. In gastrointestinal malignancies, early dysbiosis characterized by enrichment of taxa such as *Fusobacterium nucleatum* precedes tumor development and supports non-invasive diagnostic strategies (57, 133). Integration of taxonomic profiles with metabolic gene signatures increases diagnostic resolution across cancer types.

Microbial composition and function provide prognostic information through associations with tumor stage, molecular subtype, and survival. Tissue-resident taxa correlate with genomic alterations, DNA repair pathways, and transcriptional programs linked to hypoxia, immune activation, and metabolic rewiring, enabling construction of microbiome-derived risk scores independent of clinical variables. Enrichment of procarcinogenic bacteria, including *Fusobacterium nucleatum*, associates with advanced disease and reduced survival, whereas commensal-dominated communities correlate with improved outcomes and therapeutic sensitivity. Strain-resolved metagenomics improves predictive performance by capturing functional variability not detectable at species level, particularly when integrated with machine learning models. Predictive accuracy increases when microbial features are aligned with treatment regimens, indicating that microbial ecosystems reflect both tumor biology and therapy-driven selection pressures (82, 133).

Therapeutic response across immunotherapy, chemotherapy, and radiotherapy is associated with microbial regulation of immune and metabolic states within the TME. Variability in immune checkpoint blockade efficacy correlates with microbial diversity and functional capacity, influencing antigen presentation, dendritic cell activation, and CD8^+^ T cell cytotoxicity. Dysbiotic microbial configurations associated with barrier disruption correlate with inflammatory and immunosuppressive states within the TME through signaling pathways discussed in the molecular mechanisms section. In contrast, enrichment of commensal-dominated microbial communities correlates with improved immunotherapy responsiveness and reduced treatment-associated toxicity. Clinical studies demonstrate that fecal microbiota transplantation (FMT) from responders restores sensitivity to anti-PD-1 therapy, supporting a causal contribution of microbial composition to treatment efficacy. Microbial modulation through probiotics, diet, or targeted interventions further associates with variability in treatment response and toxicity profiles across therapeutic modalities (4, 134).

Microbiome–drug interactions influence pharmacokinetics and pharmacodynamics through enzymatic transformation of drugs and regulation of host metabolic states. Microbial enzymes such as β-glucuronidases deconjugate drug metabolites, altering enterohepatic circulation and systemic exposure, while bile acid–modifying microbial communities is associated with variability in systemic drug metabolism and therapeutic response. Bidirectional interactions occur as anticancer therapies reshape microbial composition, whereas microbial communities influence drug absorption, distribution, metabolism, and excretion. In breast cancer, microbiome composition has been associated with variability in estrogen metabolism, treatment responsiveness, and therapy-associated toxicity during endocrine treatment and chemotherapy. Additional evidence supports microbiome-targeted interventions, including dietary modulation, probiotics, and FMT, as strategies under investigation to improve therapeutic response and reduce resistance (135–137).

## Microbiome-targeted therapeutic strategies

Microbiome-targeted therapeutic strategies are being investigated as adjunct approaches to modulate microbial composition and function in cancer. Current evidence is derived predominantly from preclinical models, observational cohorts, and early-phase clinical studies, whereas standardized therapeutic protocols and prospective clinical validation remain limited. Ongoing translational research aims to determine how microbial community composition and function influence therapeutic response, treatment toxicity, and tumor microenvironment dynamics.

Dietary interventions represent one of the most accessible approaches for microbiome modulation. High-fiber dietary patterns have been associated with increased microbial diversity and altered metabolite profiles linked to improved responses to ICIs in observational studies. In contrast, broad-spectrum antibiotic exposure has been associated with reduced microbiome diversity and less favorable immunotherapy outcomes. Interpretation of diet–microbiome interactions remains complicated by interindividual variability, differences in dietary adherence, baseline microbiome composition, and heterogeneity across study populations (138, 139).

Probiotics, prebiotics, and postbiotics are being explored as strategies capable of modifying microbial community structure and functional activity. Experimental studies suggest potential associations with epithelial barrier maintenance, inflammatory regulation, and treatment responsiveness; however, evidence in oncology remains heterogeneous and largely preclinical. Considerable variability in bacterial strains, formulation design, dosage, treatment duration, and patient selection limits reproducibility across studies and complicates translation into standardized clinical applications (140, 141).

FMT has emerged as a potential strategy for restoring microbial diversity in patients with treatment-resistant disease, particularly in the context of immunotherapy. Early clinical studies have reported associations between FMT and partial recovery of responsiveness to ICIs in selected patients. Nevertheless, available evidence remains limited by small cohort size, donor-dependent variability, lack of standardized transplantation protocols, and uncertainty regarding long-term safety and durability of microbial engraftment (142, 143).

Mechanistically, FMT aims to re-establish microbial functions associated with effective antitumor immunity rather than simply increasing microbial diversity or replacing bacterial taxa. Successful donor microbiota engraftment has been associated with restoration of microbial metabolite production, improved dendritic cell maturation, enhanced antigen presentation, increased CD8^+^ T-cell activation, and reduced immunosuppressive cell populations. Functional remodeling of the intestinal microbiome may therefore promote systemic immune activation and improve responsiveness to immune checkpoint blockade by restoring microbial signaling pathways that support antitumor immunity (144, 145).

Clinical evidence remains encouraging but preliminary. Phase I clinical studies in patients with metastatic melanoma refractory to anti-PD-1 therapy have shown that FMT from immunotherapy responders restored clinical responses in a subset of recipients, accompanied by durable donor microbiota colonization and immune reprogramming. Similar approaches are currently under investigation in colorectal, lung, renal, and other solid tumors, although most studies remain in early clinical phases. Broader clinical implementation will require standardized donor selection, pathogen screening, transplantation protocols, long-term monitoring of donor microbiota persistence, and identification of functional microbial signatures that predict therapeutic benefit (142, 146, 147).

Engineered microbial platforms and live biotherapeutic products are being investigated as experimental approaches for localized delivery of therapeutic molecules within tumor-associated environments. Preclinical studies suggest potential applications in immune modulation, antigen delivery, and selective metabolic targeting; however, clinical translation remains at an early stage. Major limitations include biosafety control, host immune interactions, systemic dissemination risk, manufacturing reproducibility, and regulatory standardization (148, 149).

Microbiome-directed interventions increasingly emphasize modulation of microbial functional activity rather than simple taxonomic manipulation. Microbiome composition has been associated with variability in therapeutic response, treatment toxicity, and systemic drug metabolism across multiple cancer types. Most available evidence remains associative or preclinical, and clinically validated microbiome-based interventions are still lacking (78, 136).

## Limitations and methodological challenges

Cohort heterogeneity introduces substantial variability in microbiome–cancer studies due to the dependence of microbial composition on host and environmental covariates. Variables such as diet, body mass index, inflammatory status, medication exposure, and lifestyle factors account for a large proportion of microbiome variance and can exceed disease-associated effects if not explicitly modeled. Quantitative profiling demonstrates that parameters such as intestinal transit time and inflammation metrics alter microbial abundance independently of the diagnostic category, leading to loss of previously reported associations after covariate adjustment. Inter-individual variability further complicates interpretation, as distinct taxonomic profiles may converge functionally, limiting reproducibility of taxa-based biomarkers across cohorts. In predictive frameworks, insufficient control of covariates introduces bias and reduces generalizability across independent datasets (150–152).

Sampling strategies and study design generate systematic variability across microbiome datasets. Sample type selection determines the ecological niche captured and influences interpretation of host–microbe interactions, with tissue, stool, and blood samples providing non-equivalent biological information (153). Collection procedures, storage conditions, and extraction protocols introduce technical variation that can exceed biological signals. In low-biomass environments such as tumor tissues, microbial DNA approaches detection limits, increasing susceptibility to contamination from reagents, operators, and laboratory environments. Cross-contamination, well-to-well leakage, and batch effects during processing further distort compositional estimates and can confound case–control comparisons when samples are not randomized across batches. Contamination-aware workflows, negative controls, and standardized reporting are required to mitigate these biases (154, 155).

Sequencing approaches impose constraints on taxonomic resolution and functional inference. 16S rRNA gene sequencing provides limited phylogenetic resolution and does not capture functional capacity, restricting interpretation to higher taxonomic levels. Shotgun metagenomics enables species- and strain-level resolution and functional profiling but introduces challenges related to host DNA dominance, computational complexity, and database dependence. In tumor-associated datasets, host-derived sequences can be misclassified as microbial reads, generating false-positive signals when host depletion and filtering are insufficient. Incomplete or contaminated reference databases further reduce annotation accuracy and contribute to inconsistencies across analytical pipelines (156, 157).

Causal inference remains limited due to the predominance of cross-sectional and association-based designs. Microbial signatures identified in cancer cohorts may reflect secondary effects of inflammation, metabolic disruption, or treatment exposure rather than direct contributions to carcinogenesis. Longitudinal evidence across cancer types remains limited, making it difficult to distinguish whether microbiome alterations precede disease onset or primarily reflect disease progression and therapeutic exposure (158). Reverse causality and confounding-driven associations cannot be excluded in observational datasets. Analytical artifacts derived from inappropriate computational pipelines or insufficient filtering can generate disease-specific microbial signatures in the absence of biological signal, as demonstrated by reanalysis and retraction of tumor microbiome studies. Establishing causality requires longitudinal sampling, controlled perturbation experiments, and validation in model systems linking microbial features to defined host pathways (159, 160).

Integration of multi-omic datasets introduces high-dimensional analytical challenges. Combined analysis of metagenomics, metabolomics, and host-derived data generates large feature spaces with complex dependency structures. Standard approaches often yield extensive lists of associated features without capturing coordinated cross-omic interactions or biologically coherent modules. Multicollinearity, feature redundancy, and heterogeneity across omics platforms reduce interpretability and stability of inferred associations. Integration strategies based on intermediate representations or module detection improve coherence but remain difficult to standardize across datasets with differing structure and scale (161).

Reproducibility and standardization remain unresolved limitations. Differences in experimental protocols, sequencing platforms, and bioinformatic pipelines produce inconsistent results across studies, even when addressing similar clinical questions. Batch effects and processing biases introduce systematic differences when cases and controls are not balanced across experimental conditions. Machine learning models frequently exhibit overfitting, data leakage, and reduced external validity when trained on microbiome datasets with limited sample size and high dimensionality. Lack of standardized validation frameworks and independent cohort testing limits clinical translation. Community-driven efforts to establish standardized workflows and reporting guidelines exist but are not uniformly implemented (150, 162).

## Conclusions and future perspectives

The evidence synthesized in this review supports the human microbiome as a systems-level regulator of cancer biology. Microbial communities influence tumor development through coordinated interactions between microbial metabolites, structural components, enzymatic activities, and host signaling pathways that operate across immune, metabolic, neural, and endocrine systems. Although the relative contribution of each regulatory axis differs among tumor types, convergence through shared intracellular signaling networks provides a unifying framework for understanding how microbiome-derived signals influence tumor initiation, progression, and therapeutic response. This systems-level perspective extends beyond individual mechanisms by integrating molecular communication across multiple biological systems into a coherent model of host–microbiome interactions (3, 5, 163).

An important concept emerging from current evidence is that microbiome function is better explained by microbial activity than by taxonomic composition alone. Functional outputs, including metabolites, structural molecules, and microbial enzymatic processes, determine the biological consequences of host–microbiome interactions through receptor-mediated signaling and downstream intracellular pathways. Consequently, future studies should increasingly prioritize functional characterization and pathway-level analysis rather than relying exclusively on descriptive microbial profiles. Such an approach is likely to improve the biological interpretation of microbiome-associated signatures across different malignancies (58, 78).

Longitudinal and interventional study designs remain necessary to resolve temporal dynamics and establish causality. Microbiome-associated tumor effects arise from dynamic interactions involving diet, microbial metabolism, host physiology, medication exposure, and disease stage, indicating that static compositional profiling alone does not adequately capture functional biological states. Standardized cohort designs, controlled perturbation studies, and integration of environmental and clinical variables will be essential to reduce inter-study variability and define conserved microbiome-associated cancer mechanisms (90, 147).

Despite rapid progress, several challenges continue to limit clinical translation. Much of the available evidence remains based on cross-sectional associations, heterogeneous patient populations, and variable analytical methodologies, making it difficult to distinguish causal mechanisms from disease-associated microbial alterations. Standardization of sampling procedures, sequencing strategies, bioinformatic pipelines, and reporting criteria will be necessary to improve reproducibility across studies. Equally important will be the integration of longitudinal cohorts, controlled intervention studies, and complementary experimental models capable of validating microbiome-dependent mechanisms in human cancer (5, 164, 165).

Future research will also require integration of multiple layers of biological information. Combining metagenomics with metatranscriptomics, metabolomics, proteomics, spatial molecular approaches, and host genomic and immunological profiling will facilitate identification of biologically active microbial pathways and their interactions with host signaling networks. Such multidimensional analyses are expected to clarify how microbial functional states influence immune regulation, metabolic adaptation, endocrine communication, neural signaling, and therapeutic response throughout tumor evolution (166, 167).

Continued progress in the field will depend on translating mechanistic knowledge into clinically applicable strategies. Identification of reproducible microbial biomarkers together with validation of function-based therapeutic targets may improve patient stratification, prediction of treatment response, and monitoring of disease progression. Although microbiome-targeted interventions remain under active investigation, advances in mechanistic understanding and methodological standardization provide a rational foundation for future clinical development. Integrating microbial biology with molecular oncology, immunology, and systems medicine is likely to strengthen precision oncology by incorporating microbiome-derived information into cancer prevention, diagnosis, and treatment (168–170).
